# Feasibility of intensity modulated radiotherapy with involved field radiotherapy for Japanese patients with locally advanced non-small cell lung cancer

**DOI:** 10.1093/jrr/rrab063

**Published:** 2021-07-15

**Authors:** Takanori Abe, Misaki Iino, Satoshi Saito, Tomomi Aoshika, Yasuhiro Ryuno, Tomohiro Ohta, Mitsunobu Igari, Ryuta Hirai, Yu Kumazaki, Yu Miura, Kyoichi Kaira, Hiroshi Kagamu, Shin-ei Noda, Shingo Kato

**Affiliations:** Department of Radiation Oncology, International Medical Center, Saitama Medical University, 1397-1, Yamane, Hidaka, Saitama 350-1298, Japan; Department of Radiation Oncology, International Medical Center, Saitama Medical University, 1397-1, Yamane, Hidaka, Saitama 350-1298, Japan; Department of Radiation Oncology, International Medical Center, Saitama Medical University, 1397-1, Yamane, Hidaka, Saitama 350-1298, Japan; Department of Radiation Oncology, International Medical Center, Saitama Medical University, 1397-1, Yamane, Hidaka, Saitama 350-1298, Japan; Department of Radiation Oncology, International Medical Center, Saitama Medical University, 1397-1, Yamane, Hidaka, Saitama 350-1298, Japan; Department of Radiation Oncology, International Medical Center, Saitama Medical University, 1397-1, Yamane, Hidaka, Saitama 350-1298, Japan; Department of Radiation Oncology, International Medical Center, Saitama Medical University, 1397-1, Yamane, Hidaka, Saitama 350-1298, Japan; Department of Radiation Oncology, International Medical Center, Saitama Medical University, 1397-1, Yamane, Hidaka, Saitama 350-1298, Japan; Department of Radiation Oncology, International Medical Center, Saitama Medical University, 1397-1, Yamane, Hidaka, Saitama 350-1298, Japan; Department of Respiratory Medicine, International Medical Center, Saitama Medical University, 1397-1, Yamane, Hidaka, Saitama 350-1298, Japan; Department of Respiratory Medicine, International Medical Center, Saitama Medical University, 1397-1, Yamane, Hidaka, Saitama 350-1298, Japan; Department of Respiratory Medicine, International Medical Center, Saitama Medical University, 1397-1, Yamane, Hidaka, Saitama 350-1298, Japan; Department of Radiation Oncology, International Medical Center, Saitama Medical University, 1397-1, Yamane, Hidaka, Saitama 350-1298, Japan; Department of Radiation Oncology, International Medical Center, Saitama Medical University, 1397-1, Yamane, Hidaka, Saitama 350-1298, Japan

**Keywords:** locally advanced non-small cell lung cancer, intensity modulated radiotherapy, volumetric modulated arc therapy, feasibility, durvalumab

## Abstract

The feasibility of intensity modulated radiotherapy (IMRT) with involved field radiotherapy (IFRT) for Japanese patients with locally advanced non-small cell lung cancer (LA-NSCLC) remains unclear. Here we reviewed our initial experience of IMRT with IFRT for Japanese patients with LA-NSCLC to evaluate the feasibility of the treatment. Twenty LA-NSCLC patients who were treated with IMRT with IFRT during November 2019 to October 2020 were retrospectively analyzed. All patients received 60 Gy in 30 fractions of IMRT and were administered concurrent platinum-based chemotherapy. The median patient age was 71 years old and the group included 15 men and 5 women. The patient group included 2 patients with stage IIB, 11 patients with stage IIIA, 5 patients with stage IIIB, and 2 patients with stage IIIC disease. Histological diagnosis was squamous cell carcinoma in 14 patients, adenocarcinoma in 5 patients, and non-small cell lung cancer in 1 patient. The median follow-up period was 8 months. The incidence of grade 3 or greater pneumonitis was 5%, and grade 3 or greater esophagitis was not observed. None of the patients developed regional lymph node, with only recurrence reported so far. These findings indicate that IMRT with IFRT for Japanese patients with LA-NSCLC is feasible in terms of acute toxicity. Further study with a larger number of patients and longer follow-up to clarify the effect of treatment on patient prognosis is required.

## INTRODUCTION

Lung cancer remains the leading cause of cancer-related mortality worldwide [1,2]. Non-small cell lung cancer (NSCLC) is a major pathological type of lung cancer which accounts for approximately 85% of lung cancer [3]. Concurrent chemo-radiotherapy (CCRT) is the standard treatment for inoperable locally advanced NSCLC (LA-NSCLC) [4]. Recently, durvalumab as an maintenance therapy after CCRT has been proven to have good efficacy in patients with LA-NSCLC [5–7]. Radiotherapy techniques have been improved over the last decade, and high precision radiotherapy is available for various kinds of tumors [8]. Intensity modulated radiotherapy (IMRT) is a high precision radiotherapy technique that administers a conformal dose to the target volume while sparing surrounding organs at risk compared with conventional 3-dimensional conformal radiotherapy (3D-CRT) [9]. In the secondary analysis of the radiation therapy oncology group (RTOG) 0617 trial involving patients with LA-NSCLC, the incidence of grade 3 or greater radiation pneumonitis was significantly lower with IMRT compared with 3D-CRT [10]. Although there is no direct comparison of 3D-CRT and IMRT for LA-NSCLC, many retrospective studies have shown the efficacy and safety of IMRT for LA-NSCLC [11–13]. Based on these back grounds, IMRT for LA-NSCLC is increasingly performed [14]. Another change in radiotherapy treatment for LA-NSCLC is the use of involved field radiotherapy (IFRT) instead of elective nodal irradiation (ENI). With IFRT, prophylactic lymph node area was not included as a target volume while ENI includes prophylactic lymph node area as a target volume. It is reported that radiation dose of organs at risk, such as lung or esophagus, is reduced using IFRT compared with ENI, which leads to less toxicity such as pneumonitis or esophagitis [15,16]. Schild et al. demonstrated a favorable survival outcome of LA-NSCLC patients treated with IFRT compared with ENI [17]. On the basis of these findings, IMRT and IFRT have been increasingly used in Japan. However, no studies have examined the safety of IMRT with IFRT for Japanese patients with LA-NSCLC.

We started IMRT with IFRT for patients with LA-NSCLC at our institution from December 2019. Here, we report our initial experience of IMRT with IFRT for Japanese patients with LA-NSCLC with the aim of evaluating the feasibility of this treatment. We also created a virtual 3D-CRT plan on the computed tomography (CT) image of a patient treated with IMRT and compared dose-volume parameters of organs at risk.

## MATERIALS AND METHODS

### Patients

Patients with LA-NSCLC who were treated by IMRT with IFRT between December 2019 and October 2020 were retrospectively analyzed under approval from our institutional review board (reference number: 20–167). Lung cancer was histologically diagnosed by biopsy via bronchoscopy or CT-guided needle biopsy. The clinical stage was determined by multi-modal findings, including CT, magnetic resonance imaging of brain, and fluorodeoxyglucose positron emission tomography, and classified according to the Union for International Cancer Control (8th ed.). The treatment modality, such as surgery or radiotherapy, was decided by discussion at a multi-disciplinary cancer board.

### Treatment

#### Radiotherapy

Radiotherapy was performed with IMRT with 10 MV X-ray using a linear accelerator (Trilogy, Varian, California, USA). In this study, IMRT technique was volumetric modulated arc therapy (VMAT) for all the patients. For the treatment planning, CT images at mid-respiratory phase were taken. Four-dimensional CT was also taken to evaluate the tumor respiratory motion. If the tumor respiratory tumor motion was larger than 1 cm, the patient was considered ineligible for VMAT. Gross tumor volume was defined using the visible primary tumor and lymph node metastasis on CT images. Lymph node metastasis was defined as a swollen lymph node with a short axis greater than 1 cm or abnormal uptake of fluorodeoxyglucose. ENI was not performed for all the patients of this study.

Internal target volume (ITV) was created by maximum intensity projection methods using four-dimensional CT images to encompass the respiratory motion of the gross tumor volume. For the clinical target volume (CTV) margin, 0–5 mm was added to ITV. For the planning target volume (PTV) margin, 5 mm was added to CTV. Usually, 2 arcs were used and rotation angle of gantry was set to avoid contralateral lung. All treatment plans were created using Eclipse (Varian, California, USA) and calculated with AAA algorithm.

The minimum dose in the most irradiated 95% of PTV (PTV D95) should be 100% of prescribed dose. Dose constraints for organs at risk were as follows: volume of lung receiving more than 5 Gy (Lung V5) should be less than 60%, volume of lung receiving more than 20 Gy (Lung V20) should be less than 35%, maximum dose in the spinal cord should be less than 50 Gy, volume of esophagus receiving more than 60 Gy should be less than 17%, and volume of heart receiving more than 50 Gy should be less than 25%. Priority was given on dose constraints on spinal cord and lung. Volume of lung receiving more than 30 Gy (lung V30), volume of lung receiving more than 40 Gy (lung V40), volume of lung receiving more than 50 Gy (lung V50), volume of lung receiving more than 60 Gy (lung V60), volume of esophagus receiving more than 40 Gy (esophagus V40), volume of esophagus receiving more than 50 Gy (esophagus V50), volume of esophagus receiving more than 60 Gy (lung V60), volume of heart receiving more than 40 Gy (heart V40), volume of heart receiving more than 50 Gy (heart V50), and volume of heart receiving more than 60 Gy (heart V60) were also calculated and recorded.

#### Chemotherapy

All patients received platinum-based concurrent chemotherapy. Prior to chemotherapy, blood tests, including complete blood count and biochemistry tests, were performed. If necessary, echocardiograms and pulmonary function tests were also performed. The chemotherapy regimens were as follows: carboplatin plus paclitaxel, low-dose daily carboplatin, and cisplatin plus TS-1; the regimens were determined by the physician.

#### Adjuvant therapy

Durvalumab was administered as adjuvant therapy for eligible patients. Patients who developed grade 2 or greater pneumonitis or disease progression at the end of CCRT were not able to receive durvalumab. Durvalumab was intravenously administered every 3 weeks until disease progression, the development of adverse events, or 12 months from first administration.

**Table 1:** Patient and tumor characteristics (*n* = 20)

Characteristic		
Age, years, median (range)		71 (56–80)
Sex	Male	15 (75)
	Female	5 (5)
Histopathological type, n (%)	Squamous cell carcinoma	14 (70)
	Adenocarcinoma	5 (25)
	Non-small cell lung cancer	1 (5)
T classification, n (%)	T1a	1 (5)
	T1c	2 (10)
	T2a	2 (10)
	T2b	1 (5)
	T3	3 (15)
	T4	11 (55)
N classification, n (%)	N0	3 (15)
	N1	7 (35)
	N2	7 (35)
	N3	3 (15)
Clinical stage, n (%)	IIB	2 (10)
	IIIA	11 (55)
	IIIB	5 (25)
	IIIC	2 (10)
Regimen of chemotherapy, n (%)	Carboplatin+paclitaxel	11 (55)
	low dose carboplatin	8 (40)
	Cisplatin+S1	1 (5)
Tumor proportion score, n (%)	<1	7 (35)
	1–10	4 (20)
	11–50	2 (10)
	51–90	3 (15)
	> 90	1 (5)

#### Evaluation

For patients who received durvalumab, chest X-ray, complete blood cell count, and biochemistry tests were checked at every patient visit when receiving treatment. For patients who did not receive durvalumab, chest X-ray and blood test were performed monthly. In addition, chest CT was taken every 3 months or at the timing of possible progression of disease or possible pneumonitis. Diagnosis of pneumonitis was carefully determined by discussion between the radiation oncologist and respiratory medicine physicians along with multimodal examination results to exclude the possibility of bacterial or viral pneumonia. Patients who did not receive durvalumab were also periodically followed up by chest X-ray, chest CT, and blood test. Toxicities were classified using the National Cancer Institute Common Toxicity Criteria for Adverse Events, version 5.0.

#### Dosimetric comparison between VMAT and virtual 3D-CRT plan

We created a virtual 3D-CRT plan on the planning CT image for VMAT and compared dose-volume parameters such as PTV D95, lung V5, lung V20, lung V30, lung V40, lung V50, lung V60, esophagus V40, esophagus V50, esophagus V60, heart V40, heart V50, and heart V60 between VMAT and the virtual 3D-CRT plan. All the structures in the virtual 3D-CRT plan were the same as the VMAT plan. In the virtual 3D-CRT plan, beams with anterior–posterior directions and orthogonal direction to avoid the spinal cord were used. A 60 Gy in 30 fractions was prescribed to cover the 100% of PTV as VMAT plan. The isocenter of the beam was set on the soft tissue or lymph node at mediasternum, not on the air in lung parenchysema. Treatment plans were created using Eclipse (Armonk, NY, USA) and the dose calculation algorithm was AAA.

### Statistical analysis

Differences of means of parameter between two groups were compared with Student’s t-test. *P* < 0.05 was considered significant. All statistical analyses were performed using IBM SPSS Statistics for Windows, Version 25.0 (SPSS Inc., Armonk, NY, USA).

## RESULTS

### Patients and treatments

A total of 20 LA-NSCLC patients who were treated by VMAT with IFRT were retrospectively analyzed. The patient characteristics are listed in Table 1. The median patient age was 71 years old, and the study group included 15 men and 5 women. The patient group included 2 patients with stage IIB, 11 patients with stage IIIA, 5 patients with stage IIIB, and 2 patients with stage IIIC disease. Histological diagnosis was squamous cell carcinoma in 14 patients, adenocarcinoma in 5 patients, and non-small cell lung cancer in 1 patient. The radiotherapy dose was 60 Gy in 30 fractions in all patients. The chemotherapy regimen was carboplatin plus paclitaxel in 11 patients, daily low-dose carboplatin in 8 patients, and cisplatin plus TS-1 in 1 patient. At the same period of this study, 7 patients were treated with 3D-CRT. Reasons why these patients were not treated with VMAT were need for immediate start of treatment in 4 patients, uncontrolled pleural effusion in 2 patient and difficulty in resting without motion in 1 patient.

**Fig. 1.**
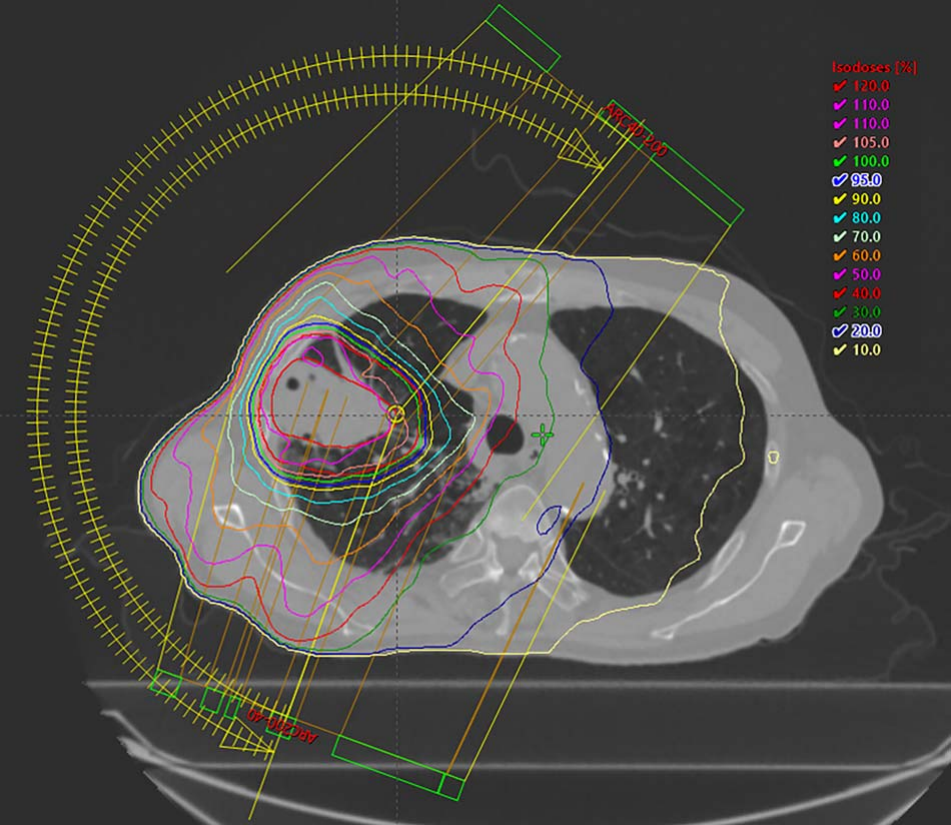
Representative treatment plan from a patient is shown. The patient had cT3N2M0 lung adenocarcinoma. The prescribed dose was 60 Gy in 30 fractions, and the blue line showed 95% of the prescribed dose.

**Fig. 2.**
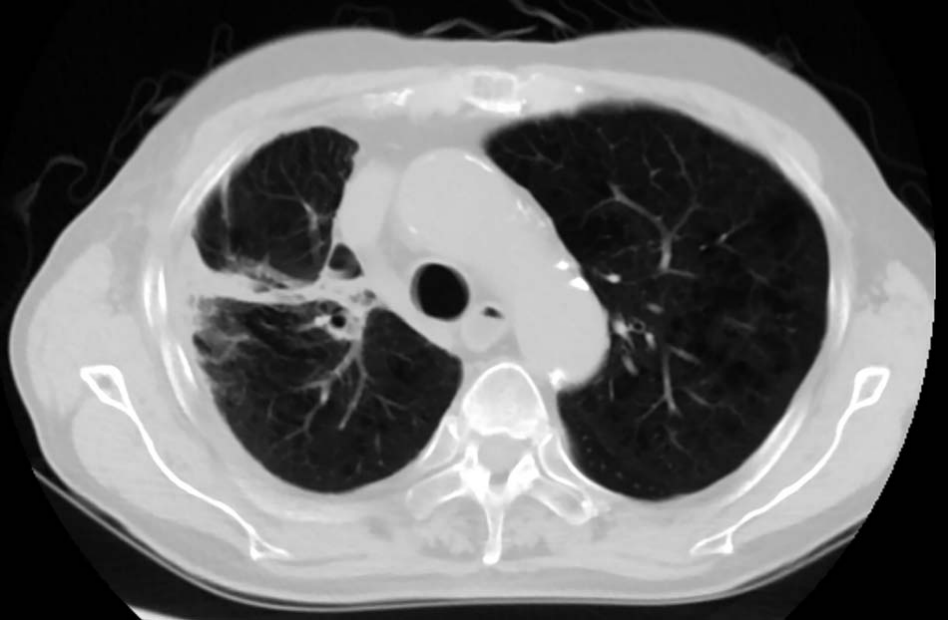
Follow-up computed tomography image of the same patient shown in Figure 1. Imaging was acquired 6 months from treatment. There was no evidence of recurrence and out-of-irradiated-field pneumonitis.

Among the 20 patients, 4 patients did not receive durvalumab. The reasons for not receiving durvalumab were patient refusal (two patients), poor general condition (one patient), and progression of disease at the end of CCRT (one patient). Among the 16 patients who received durvalumab, 13 patients had to pause or stop durvalumab treatment; 7 patients resumed administration of durvalumab and 6 patients stopped treatment entirely. The reasons for cancellation of durvalumab were pneumonitis (nine patients), progression of disease (two patients), hyperthyroidism (one patient), and thrombocytopenia (one patient). The median number of cycles of durvalumab was 6. Representative imaging results from one patient is shown in Figure 1 and 2.

### Treatment efficacy

The median follow-up period was 8 months (range: 6–15). At the last follow-up, seven patients experienced progression of disease. The patterns of progression were multiple liver metastasis plus primary tumor recurrence (one patient), bone metastasis plus primary tumor recurrence (three patients), contra-lateral lung metastasis (one patient), multiple bone metastasis (one patient) and lymph node recurrence plus pleural effusion and primary tumor recurrence (one patient). One patient died of multiple metastasis and the others were alive at the last follow-up.

### Toxicities

Grade 1 pneumonitis was observed in 11 patients (55%), grade 2 pneumonitis was observed in 6 patients (30%), and grade 4 pneumonitis was observed in 1 patient (5%). Grade 1 esophagitis was observed in 4 patients (20%) and grade 2 esophagitis was observed in 1 patient (5%). Grade 4 pneumonitis developed 3 weeks after CCRT. Patient with grade 4 pneumonitis received steroid pulse therapy and was improved after gradual decrease of steroid. Median interval between CCRT and develop of grade 2 pneumonitis was 2.5 months among 6 patients. Among them, 3 patients received predonisolone of 0.5 mg/kg and other 3 patients received cough suppressant and antipyretic. Lung V5 was 44% and lung V20 was 9% for patient with grade 4 pneuonitis. Among 6 patients with grade 2 pneumonitis, mean lung V5 was 46% and mean lung V20 was 18%.

### Dosimetric comparison between the VMAT and virtual 3D-CRT plans

Mean lung V5 in VMAT was significantly higher than that in 3D-CRT, while mean lung V30, V40, V50, and V60 were significantly lower in VMAT than in 3D-CRT. Mean lung V20 was not significantly different between VMAT and 3D-CRT. Mean esophagus V40, V50, and V60 were not significantly different between VMAT and 3D-CRT. Mean heart V40, V50, and V60 of heart were significantly lower in VMAT than that in 3D-CRT. These results are summarized in Table 2.

**Table 2:** Comparison of dosimetric parameters between VMAT and virtual 3D-CRT

Parameter	VMAT	3D-CRT	*p*- value
Lung V5, % (±SD)	47.8 (±8.6)	29.8 (±7.4)	< 0.01
Lung V20, % (±SD)	18.2 (±4.8)	20.3 (±5.5)	0.221
Lung V30, % (±SD)	11.6 (±3.7)	17.7 (±5.0)	< 0.01
Lung V40, % (±SD)	7.4 (±2.9)	15.5 (±9.1)	0.01
Lung V50, % (±SD)	4.7 (±2.1)	11.1 (±4.3)	< 0.01
Lung V60, % (±SD)	2.1 (±1.3)	7.3 (±3.5)	< 0.01
Esophagus V40, % (±SD)	7.2 (±10.7)	7.3 (±11.9)	0.993
Esophagus V50, % (±SD)	2.7 (±6.5)	3.8 (±8.8)	0.687
Esophagus V60, % (±SD)	1.0 (±3.0)	1.5 (±6.5)	0.781
Heart V40, % (±SD)	3.0 (±4.5)	8.9 (±9.5)	0.02
Heart V50, % (±SD)	1.6 (±2.4)	6.2 (±7.2)	0.014
Heart V60, % (±SD)	0.9 (±1.4)	4.4 (±5.8	0.016

VMAT: volumetric modulated arc radiotherapy; 3D-CRT: three-dimensional conformal radiotherapy; SD: standard deviation

## DISCUSSION

Here we reported the feasibility of IMRT with IFRT for Japanese patients with LA-NSCLC. In total patient group, the incidence of grade 3 or greater pneumonitis was 5%, and grade 3 or greater esophagitis was not observed. On the basis of our results, we think that IMRT with IFRT is feasible for Japanese patients with LA-NSCLC regarding acute and sub-acute toxicities. A longer follow-up with a larger number of patients is necessary to confirm these findings.

The incidence of grade 3 or greater pneumonitis in our study group was 5%, which was comparable to the previously reported incidence of 6%–12% for grade 3 or greater pneumonitis after CCRT with 3D-CRT for LA-NSCLC [10,18–20]. Among the 20 patents in our study, 16 received durvalumab, and the incidence of grade 3 or greater pneumonitis was 5% in these 16 patients. In the phase III trial of durvalumab as consolidation therapy after CCRT for LA-NSCLC (the Pacific trial), the incidence of grade 3 or greater pneumonitis was 4% [5]. We previously reported an incidence of 8% for pneumonitis after CCRT with 3D-CRT followed by durvalumab for LA-NSCLC [21]. The incidence of pneumonitis in our patients treated with IMRT with IFRT followed by durvalumab was also comparable to the results of the Pacific trial and our previous study with 3D-CRT.

In this study, V5 of lung was significantly higher in IMRT compared with the 3D-CRT plan, while V30–60 were significantly lower in IMRT. The effect of the increase of lung V5 in IMRT on the incidence of pneumonitis is unclear. Meng et al. reported that an intermediate dose irradiated volume of the lung was a significant factor to predict pneumonitis compared with a low dose irradiated volume of lung [22]. We did not observe an increase of pneumonitis with IMRT, which supports the conclusion of Meng et al. However, a longer follow-up with a larger number of patients is necessary to clarify the effect of the increase of V5 and decrease of V30–60 on the incidence of pneumonitis after IMRT for LA-NSCLC patients.

We did not observe grade 3 or greater esophagitis in this study. In contrast, other studies reported an incidence of 6%–35% for grade 3 or greater esophagitis in CCRT using 3D-CRT [10,18–20]. We speculate that the difference in incidence was because of the use of IFRT in our study, not from IMRT. In this study, dose-volume parameters of esophagus were not significantly different between the IMRT plan and the simulated 3D-CRT plan. This tendency was also observed in the secondary analysis of the RTOG0617 trial [23]. In that analysis, dose-volume parameters of esophagus were not significantly different between the 3D-CRT group and IMRT group, and the incidence of esophagitis was not also significantly different between the two groups. In contrast, the comparison of ENI and IFRT for LA-NSCLC showed that the incidence of grade 3 or greater esophagitis in the IFRT group was almost half of the incidence observed in the ENI group [15,16]. We speculate that the use of IFRT may markedly reduce the incidence of esophagitis.

In this study, the dose to the heart was significantly lower in IMRT compared with simulated 3D-CRT. In the RTOG0617 trial, the increase of the dose to the heart might be the cause of the poor prognosis in the higher dose regimen cohort [23]. Evidence has been accumulating regarding the relationship between radiotherapy dose to the heart, cardiac toxicity, and patient prognosis [24]. IMRT has advantages compared with 3D-CRT regarding dosimetric parameters to the heart, but a further study to observe patients over the long-term is necessary to accurately determine the effect of the reduction of heart dose on cardiac toxicity and patient prognosis.

One of the concerns in performing IFRT for LA-NSCLC is the increase of regional lymph node recurrence. In the current study, four patients experienced disease progression; however, none of the patients showed solitary regional lymph node recurrence. Fernandes et al. reported that the use of IFRT did not significantly increase regional lymph node recurrence [15]. We will carefully follow up our patients to clarify whether IFRT may increase regional lymph node recurrence.

This study has several limitations. First, the relatively small number of patients might cause bias on baseline patient characteristics. Second, the short follow-up period might result in an underestimation of adverse events and recurrence. However, at the least, our study provides results for acute toxicity, and the results on the reduction of acute toxicity such as esophagitis are meaningful for patient quality of life.

In conclusion, our findings indicate that IMRT with IFRT for Japanese patients with LA-NSCLC is feasible in terms of acute toxicity. A further study with larger patients and longer follow-up is necessary to clarify its effect on actual prognosis of patients.

## Abbreviations

IMRT: intensity modulated radiotherapy; IFRT: involved field radiotherapy; LA-NSCLC: locally advanced non-small cell lung cancer; CCRT: concurrent chemoradiotherapy; 3D-CRT: three-dimensional radiotherapy; RTOG: radiation therapy oncology group; ENI: elective nodal irradiation; CT: computed tomography; VMAT: volumetric modulated arc therapy; FDG: fluorodeoxyglucose; GTV: gross tumor volume; ITV: internal target volume; CTV: clinical target volume; PTV: planning target volume; lung V5: volume of lung receiving more than 5 Gy; lung V20: volume of lung receiving more than 20 Gy; lung V30: volume of lung receiving more than 30 Gy; lung V40: volume of lung receiving more than 40 Gy; lung V50: volume of lung receiving more than 50 Gy; lung V60: volume of lung receiving more than 60 Gy; esophagus V40: volume of esophagus receiving more than 40 Gy; esophagus V50: volume of esophagus receiving more than 50 Gy; esophagus V60: volume of esophagus receiving more than 60 Gy; heart V40: volume of heart receiving more than 40 Gy; heart V50: volume of heart receiving more than 50 Gy; heart V60: volume of heart receiving more than 60 Gy.

## Presentation at a conference

None.
